# Human Papillomavirus Prevalence in a Population of Women Living in Port-au-Prince and Leogane, Haiti

**DOI:** 10.1371/journal.pone.0076110

**Published:** 2013-10-03

**Authors:** David K. Walmer, Paul S. Eder, Laura Bell, Hiam Salim, Lori Kobayashi, Jackie Ndirangu, Nicole Tinfo, Philip E. Castle

**Affiliations:** 1 Family Health Ministries, Durham, North Carolina, United States of America; 2 Duke Global Health Institute, Durham, North Carolina, United States of America; 3 Qiagen Sciences, Inc., Gaithersburg, Maryland, United States of America; 4 Global Cancer Initiative, Chestertown, Maryland, United States of America; IPO, Inst Port Oncology, Portugal

## Abstract

**Background:**

There have been no published studies of carcinogenic human papillomavirus (HPV)--the necessary cause of cervical cancer--in Haiti, a nation that has one of the greatest burdens of cervical cancer globally.

**Objective:**

Characterize prevalence of carcinogenic HPV and the prevalence of individual carcinogenic HPV genotypes in women with cervical precancer or cancer, cervical intraepithelial neoplasia grade 2 (CIN2) or more severe (CIN2+).

**Methods:**

Women (n=9,769; aged 25-60 years) were screened for carcinogenic HPV by Hybrid Capture 2 (HC2; Qiagen, Gaithersburg, MD). Carcinogenic HPV positives underwent colposcopy and visible lesions were biopsied. A subset of carcinogenic HPV positives was tested for individual HPV genotypes using a GP5+/6+ assay.

**Results:**

The prevalence of carcinogenic HPV was 19.0% (95% confidence interval: 18.4%-19.9%) and decreased with increasing age (p_trend_ < 0.001). Women with 3 or more sexual partners and who started sex before the age of 18 years had twice the age-adjusted prevalence of carcinogenic HPV of women with one partner and who started sex after the age of 21 (24.3% vs. 12.9%, respectively). HPV16 and HPV35 were the most common HPV genotypes detected in CIN2+ and more common in women with CIN2+ than those without CIN2+. HPV16 and/or HPV18 were detected in 21.0% of CIN2 (n = 42), 46.2% of CIN3 (n = 52), and 80% of cancers (n = 5).

**Conclusions:**

The prevalence of carcinogenic HPV in Haiti was much greater than the prevalence in other Latin American countries. High carcinogenic HPV prevalence and a lack of cervical cancer screening may explain the high burden of cervical cancer in Haiti.

## Introduction

Cervical cancer is a major cause of cancer mortality in women of Haiti. In an early study of cancers in Haiti [1], cervical cancer represented 40% of all female cancers. Some estimates have put the cervical cancer annual incidence (93 per 100,000) and mortality (61.1 per 100,000) to be among the highest in the world [2] and perhaps the highest in Latin America [3], although estimates may be imprecise due to the quality of data collected there [3]. Regardless of the exact estimate, it is generally accepted that in Haiti incidence and mortality due to cervical cancer is very high.

Among the reasons for high incidence of and mortality from cervical cancer in Haiti is a lack of widespread screening for cervical precancer, cervical intraepithelial neoplasia grade 2 (CIN2) or CIN3, by cervical cytology (Papanicolaou test) or visual inspection with acetic acid with or without magnification (VIAM or VIA). Indeed, regular health exams are rare among the population. Furthermore, even with examinations once or a few times in a lifetime, the accuracy and quality of such exams can be compromised due to lack of quality infrastructure, medical review, quality control, and aggressive follow-up for intervention. Also, chronic inflammation in Haitian women who have never been screened can compromise an effective diagnosis from slide smears (D. Walmer, personal communication).

Since the discovery of human papillomavirus (HPV) in cervical cancer by Harold Zur Hausen (2008 Nobel Laureate in Medicine) and colleagues more than 30 years ago [4,5], there have been rapid advances in our understanding of the cause of cervical cancer. We now know that persistent cervical infections by certain types of HPV, designated as high-risk, carcinogenic, or cancer-associated, cause virtually all cervical cancer everywhere in the world [6]. HPV also causes a significant number of vulvar, vaginal, anal, penile, and oropharyngeal cancers [7]. Approximately 5% of the human burden of cancer is caused by HPV [7]. HPV16 is the most important HPV genotype, responsible for 60% of cervical cancer cases [8]. HPV18 is the next most important HPV genotype, responsible for 10% of cervical cancer, including 30% of adenocarcinoma of the cervix [8], which is on the rise in some western countries [9,10]. Together, HPV16 and 18 account for 70% of cervical cancer and a greater proportion of HPV-positive cancers of other epithelia [11].

The discovery of a single, necessary cause of cervical cancer has led to the development of prophylactic HPV vaccines for primary cancer prevention of HPV infections and molecular testing for carcinogenic HPV for secondary cancer prevention through the early detection of cervical precancer and cancer. Both have demonstrated high degrees of efficacy with maximum *population* effectiveness guided by an understanding of the causal model of cervical carcinogenesis and by an application of these technologies in an age-appropriate manner relative to the exposure to HPV [6].

Using a comprehensive search strategy, our research has found that no detailed study on HPV prevalence in Haiti has been published to date, despite the importance that such data can provide for planning effective primary and secondary prevention programs to reduce the prevalence of cervical cancer [6]. As part of a routine women’s health exam, we screened almost 10,000 women aged 25-60 years for carcinogenic HPV from 2007-2010 who were entering clinics in Leogane and Port-au-Prince, Haiti. Women were screened for carcinogenic HPV and those whose cervical specimens tested positive underwent colposcopy and visible lesions were biopsied. A subset of carcinogenic HPV positives was tested for individual HPV genotypes. Here we report the prevalence of carcinogenic HPV overall and by age, and the relationship of individual carcinogenic HPV genotypes to the diagnosis of CIN2+.

## Methods

### Ethics Statement

This study was approved by the Western IRB (Seattle, WA, USA) and by the Misyon Sante Fanmi Ayisyen IRB in Haiti.

### Study Location and Population

This study was conducted in two medical centers situated in the cities of Port-au-Prince and Leogane, Haiti. Women attending two cervical-cancer prevention clinics established by Family Health Ministries were invited to participate. Recruitment was done by word-of-mouth and local radio announcements. Women were eligible to participate if they (a) were aged 25 to 60 years of age, (b) reported having at least one sexual partner in their lifetime, (c) were not pregnant, (d) were not menstruating at time of screening, and (e) had not had a hysterectomy. A total of 9,771 women who met these eligibility criteria were enrolled.

Trained personnel obtained written informed consent from all eligible women. The consent forms were translated into Haitian Kreyol, and bilingual personnel facilitated the consenting process. Each participant was required to sign the consent form to signify comprehension; illiterate participants provided consent by placing an X on the consent form. A subject ID number was assigned to identify each specimen. To ensure confidentiality, no other participant-identifiers such as demographic information were used outside the specimen collection site. 

### Data and Specimen Collection

Trained personnel using a questionnaire designed to collect information on socio-economic demographics, reproductive health information, and contraceptive use interviewed eligible and consenting participants. Cervical samples were collected using the *digene* ^®^ cervical brush (Qiagen, Gaithersburg, MD, USA), suspended in Specimen Transport Medium™ (STM) and stored at ambient temperature before being sent to Qiagen^®^ for HPV testing.

### HPV-DNA Testing

One day after collection specimens were shipped at ambient temperature and stored at 4°C at Qiagen until ready for testing (within seven days of collection, adhering to the package insert). Specimens were tested for the presence of one or more of a pool of carcinogenic HPV (HPV16, 18, 31, 33, 35, 39, 45, 51, 52, 56, 58, 59, and 68) using Hybrid Capture 2 (HC2; Qiagen) according to the package insert. 

A subset of HC2-positive specimens was tested for 49 individual HPV genotypes (HPV2, 3, 6, 7, 10, 11, 13, 16, 18, 26-28, 30-33, 35, 39, 40, 42, 45, 51-56, 58, 59, 61, 62, 66-70, 72-74, 81-87, and 89-91) using a modified version of GP5+/6+ PCR amplification and detection assay [12].This test had been validated in-house through a third-party reference-test proficiency panel and assessment as part of the 2010 WHO LabNet study for validating in-house HPV genotyping tests [13]. 

DNA was extracted from a 100-µL aliquot of the cervical specimen using the Qiagen QIAamp® Media MDx Kit according to the recommended protocol. The DNA was eluted from the columns with 100 µL of buffer AVE (RNase-free water containing 0.04% sodium azide) from the kit. A volume of 10 µL of extracted material was used in a 50-µL PCR reaction.

The HPV genotyping results were classified hierarchically according to cancer risk [8,14]: HPV16 positive, else HPV16 negative and HPV18 positive, else HPV16 and HPV18 negative and positive for other carcinogenic HPV genotypes (HPV31, 33, 35, 39, 45, 51, 52, 56, 58, 59, or 68), else negative for all carcinogenic HPV genotypes and positive for non-carcinogenic HPV genotypes, else no HPV genotype detected.

#### Colposcopy and Management

Women who tested HC2 positive underwent routine colposcopy. Visible, acetowhite lesions were biopsied. Biopsies were read by a single pathologist at Hopital Sainte Croix in Leogane, Haiti. Women with CIN2/3 were treated with cryotherapy or hysterectomy based on clinical judgment. Women with cancer were referred to the General Hospital in Port-au-Prince or to Groupe de Support Contre Le Cancer, a support group in Haiti dedicated to cancer prevention and education.

#### Statistics

Of the 9,771 women enrolled, 9769 (99.9%) had HC2 results available and subsequent analyses were restricted to this group. Age-group (five-year age groups from 25 to 54 and a final age group of 55-60) specific prevalence of carcinogenic HPV as detected by HC2 with binomial 95% confidence intervals (95% CI) was calculated. Pearson chi-square, Fisher’s exact tests, and/or a non-parametric test of trend [15] were used to test the association of socio-demographic variables with testing carcinogenic HPV positive. A logistic regression model was used to estimate the age-adjusted prevalence of carcinogenic HPV and 95%CI for subgroups defined by categories of age at first sex (years) and lifetime number of sexual partners. Pearson chi-square, Fisher’s exact tests, and/or a non-parametric test of trend [15] were also used to test for the statistical differences between groups for detection of individual carcinogenic HPV genotypes. A p value of <0.05 was considered statistically significant. Stata version12.1 (College Station, TX) was used for all analyses.

## Results

**Figure 1:**
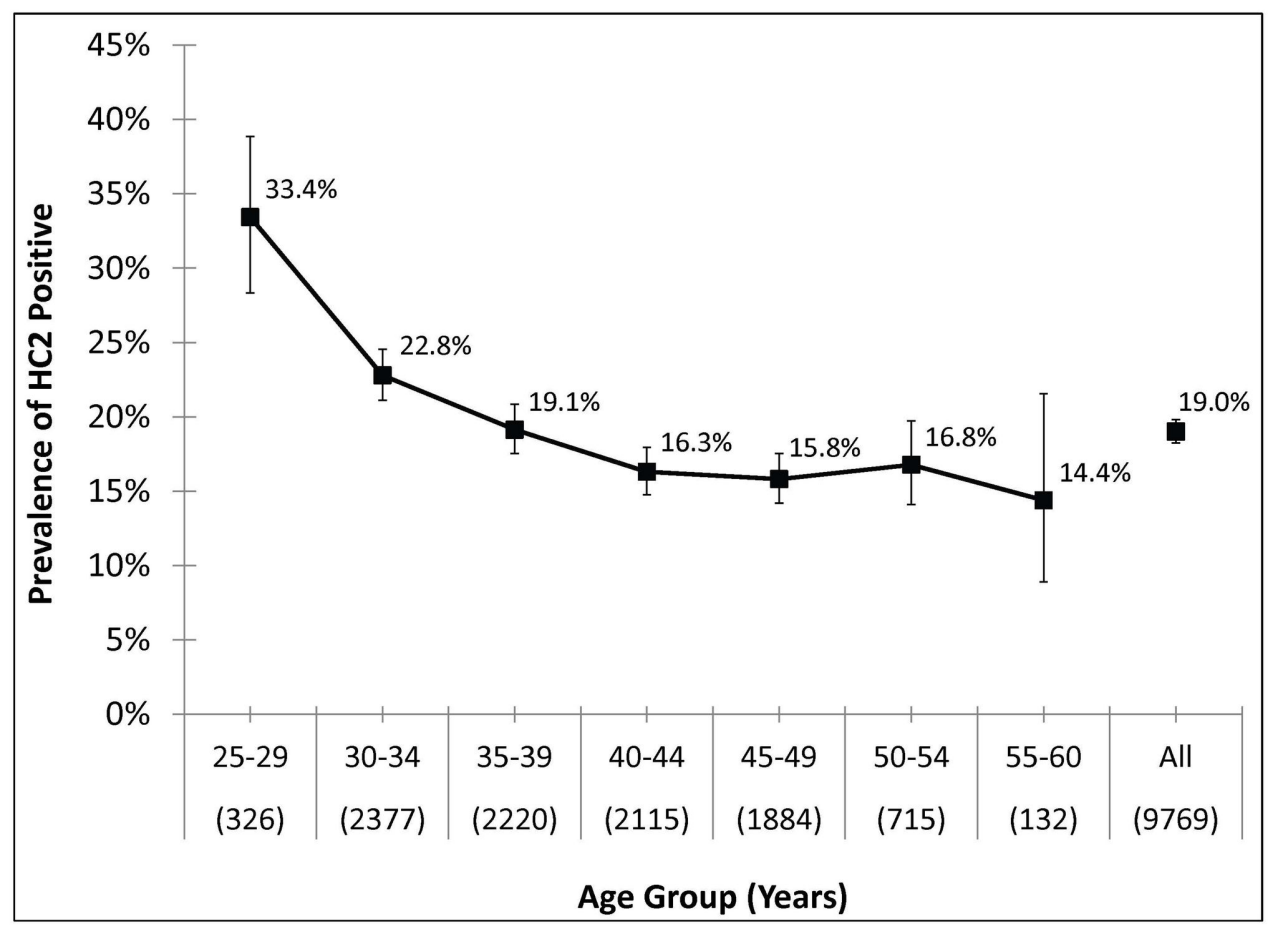
Carcinogenic HPV prevalence by age range. The age-group specific prevalence of carcinogenic human papillomavirus (HPV) DNA as detected by Hybrid Capture 2 (HC2). Bars represent 95% confidence intervals .

The overall prevalence of carcinogenic HPV in the 9,769 women tested by HC2 was 19.0% (95%CI: 18.2-19.9%) (Figure **1**). The prevalence of HR-HPV declined with age (p_trend_ < 0.001), from 33.4% (95%CI: 28.5-38.8%) in women aged 25-29 years of age to 14.4% (95%CI: 8.9%-21.7%) in women aged 55-60 years of age.

Table **1** shows the socio-demographics of this population and their relationship to testing HC2 positive. Most women were Protestant (56.8%), married (50.2%), reported two or more sexual partners in their lifetime (60.4%), and had two or more pregnancies (66.4%). The age median, mean, and range were 39, 39.7, and 25-60 years. The ages at first sex median, mean, and range were 19, 20.1, and 6-43 years. The ages at first menses median, mean, and range were 15, 14.6, and 8-28 years. The ages at first pregnancy median, mean, and range were 22, 22.9, and 10-49 years.

**Table 1:** Population Demographics.

		**All**		**HC2+**		**p**
		**n**	**%**	**n**	**%HC2+**	
**Religion**						
	Catholic	2,659	26.7%	604	22.7%	<0.001
	Christian	1,262	12.7%	189	15.0%	
	Protestant	5,661	56.8%	1,019	18.0%	
	Other	162	1.6%	28	17.3%	
	None	88	0.9%	26	29.5%	
	Missing	131	1.3%	43	32.8%	
**Marital Status**						
	Single/Living Together	4,397	44.1%	1,079	24.5%	<0.001
	Married	4,982	50.0%	696	14.0%	
	Divorced/Widowed	454	4.6%	93	20.5%	
	Other/Missing	130	1.3%	41	31.5%	
**Age at First Sex**						
	<18	2,609	26.2%	595	22.8%	<0.001
	18-19	2,203	22.1%	427	19.4%	
	20-22	2,104	21.1%	374	17.8%	
	23 and older	2,184	21.9%	320	14.7%	
	Missing	863	8.7%	193	22.4%	
**Number of Sex Partners, Lifetime**						
	1	3,895	39.1%	572	14.7%	<0.001
	2	3,023	30.3%	621	20.5%	
	3 or more	2,936	29.5%	673	22.9%	
	Missing	109	1.1%	43	39.4%	
**Parity**						
	0	1,521	15.3%	264	17.4%	<0.001
	1	1,824	18.3%	372	20.4%	
	2	2,073	20.8%	392	18.9%	
	3	1,794	18.0%	348	19.4%	
	4-5	1,941	19.5%	366	18.9%	
	6+	742	7.4%	135	18.2%	
	Missing	68	0.7%	32	47.1%	

Demographics of the population (n = 9,769) screened in Port-au-Prince and Leogane, Haiti, overall and stratified on Hybrid Capture 2 (HC2) results

Religion, marital status, age at first sex (categorical), lifetime number of sexual partners, and parity were all associated with testing HC2 positive (p < 0.001 for all). Notably, there was a trend of increasing likelihood of testing HC2 positive with increasing numbers of sexual partners and decreasing likelihood of testing HC2 positive with older age at first sex (p_trend_ < 0.001).

We used a logistic regression model to estimate the age-adjusted carcinogenic HPV prevalence (as detected by HC2) by lifetime number of sexual partners (1, 2, 3 or more, and missing/unreported) and category of age at first sex (13-17, 18-19, 20-21, 22 years and older, and missing/unreported) (Table **2**). Of those with reported values for sexual behaviors, prevalence ranged from a low of 12.9% (95%CI = 11.5%-14.4%) for women who self-reported only one sex partner and who started sex at the age of 22 years or older (n = 1,493) to a high of 24.3% (95%CI = 22.3%-26.3%) for those who self-reported 3 or more sex partners and who started sex before age 18 years (n = 1,295). Those with missing/unreported values for sexual behaviors tended to have higher prevalence than those who reported values.

**Table 2:** HPV prevalence by number of partners and age of first sex.

			**Age at First Sex (Years)**					
			**13-17**	**18-19**	**20-21**	**≥22**	**Missing**	**All**
**Number of Sex Partners (Lifetime)**	**1**	n	515	664	873	1,493	294	3,839
		%HC2+	17.1%	15.0%	14.4%	12.9%	16.1%	14.9%
	**2**	n	749	746	713	466	292	2,966
		%HC2+	22.7%	20.0%	19.4%	17.6%	22.1%	20.2%
	**3 or more**	n	1,265	754	479	195	195	2,888
		%HC2+	24.3%	21.8%	21.3%	18.8%	24.0%	22.0%
	**Missing**	n	25	7	8	6	30	76
		%HC2+	32.3%	27.9%	26.6%	23.7%	30.9%	28.2%
	**All**	n	2,554	2,171	2,073	2,160	811	9,769
		%HC2+	19.9%	18.1%	18.1%	16.8%	20.8%	18.5%

Carcinogenic human papillomavirus (HPV) prevalence, as detected by Hybrid Capture 2, stratified by lifetime number of sexual partners and age of first sex.

Finally, among HC2 positives (n =1,858), 1,608 (86.5%) were tested for HPV genotypes and 1,252 of the 1,608 (77.9%) had at least one detectable HPV genotype (Table **3**). Women with a diagnosis of <CIN2 were more likely to have a missing HPV genotyping result than women with a diagnosis of CIN2+ (22.7% vs. 15.4%, p = 0.08). There was no significant trend with age for any HPV genotype among HC2 positives with HPV genotyping (data not shown).

**Table 3:** Individual carcinogenic HPV genotypes.

	All Women		Women with CIN2+	
	All	Single	All	Single
	n = 1,252	n = 882	n = 99	n = 81
HPV16	15.8%	15.7%	26.3%	25.9%
HPV18	10.9%	10.8%	11.1%	9.9%
HPV31	8.7%	8.4%	10.1%	11.1%
HPV33	5.8%	5.3%	7.1%	7.4%
HPV35	12.2%	11.2%	19.2%	19.8%
HPV39	3.8%	3.1%	3.0%	0.0%
HPV45	9.0%	7.4%	4.0%	2.5%
HPV51	6.8%	5.8%	1.0%	1.2%
HPV52	14.7%	13.4%	13.1%	12.4%
HPV56	5.1%	4.0%	1.0%	1.2%
HPV58	4.6%	4.1%	8.1%	6.2%
HPV59	3.7%	3.1%	1.0%	0.0%
HPV68	9.4%	7.9%	4.0%	6.2%

Individual carcinogenic human papillomavirus (HPV) genotypes detected in the 1,252 women with any genotyping result, 882 women with a single carcinogenic HPV genotype detected, 99 women with a cervical intraepithelial neoplasia grade 2 (CIN2) or more severe (CIN2+) diagnosis, and 81 women with CIN2+ diagnosis and a single carcinogenic HPV genotype detected.

Of the 1252 confirmed HR-HPV positives (that is, positive both by HC2 and by PCR genotyping), 1198 available patients were referred for colposcopy (Table **4**). Most of these women did not require a biopsy (818 of 1198). From the sub-group of 380 who were biopsied, 99 (26.1%) were diagnosed as CIN2+. Therefore, among 1198 patients with confirmed HR-HPV, 8.3% (99/1198) were diagnosed as CIN2+.

**Table 4:** The relationship of hierarchical HPV status and histologic diagnosis.

	Total		Missing		No Biopsy†		Neg		CIN1		CIN2		CIN3		CxCa		<CIN2*		CIN2+	
**HPV Status among HC2+**	n	%	n	%	n	%	n	%	n	%	n	%	n	%	n	%	n	%	n	%
HPV16	198	15.8%	7	13.0%	133	16.3%	14	11.3%	18	11.5%	6	14.3%	18	34.6%	2	40.0%	172	14.9%	26	26.3%
HPV18	132	10.5%	8	14.8%	88	10.8%	15	12.1%	10	6.4%	3	7.1%	6	11.5%	2	40.0%	121	10.5%	11	11.1%
Other Carcinogenic	783	62.5%	34	63.0%	508	62.1%	76	61.3%	109	69.4%	30	71.4%	25	48.1%	1	20.0%	727	63.1%	56	56.6%
Non-Carcinogenic	139	11.1%	5	9.3%	89	10.9%	19	15.3%	20	12.7%	3	7.1%	3	5.8%	0	0.0%	133	11.5%	6	6.1%
Total	1252	100%	54	100%	818	100%	124	100%	157	100%	42	100%	52	100%	5	100%	1153	100%	99	100%

The relationship between hierarchical human papillomavirus (HPV) status (HPV16 > HPV18 > carcinogenic HPV other than HPV16&HPV18 > non-carcinogenic) and histologic diagnosis. Those without HPV genotyping result because it was not done or no specific type was detected are shown. The % is the column percentage among those with genotyping results. Biopsies were only done among those who were referred to colposcopy because of a positive Hybrid Capture 2 result (HC2+) and had visible lesions.

*Includes women who had missing biopsy result or no biopsy taken

† Went to colposcopy but no visible lesion was observed and therefore no biopsy was taken

Of those who tested positive for HPV genotypes, 231 of 1,252 (18.5%) had multiple carcinogenic HPV genotypes detected. HPV16 was the most common carcinogenic HPV genotype detected overall as well as among those with a single carcinogenic HPV genotype detected, and for all women diagnosed with CIN2+. Among women with CIN2+ diagnoses and single carcinogenic HPV infections detected, HPV16 (25.9%), HPV35 (19.8%), HPV52 (12.4%), HPV31 (11.1%), and HPV18 (9.9%) were the most common HPV genotypes detected whereas HPV39 and HPV59 were not detected. HPV16 (p = 0.006 for all, p = 0.015 for single infections) and HPV35 (p = 0.036 for all, p = 0.016 for single infections) were more common in women with CIN2+ than without CIN2+. HPV18, 31, 33, 52, 58, and 68 were equally common in women with CIN2+ as without CIN2+. HPV39, 45, 51, 56, and 59 were less common in women with CIN2+ than without CIN2+ (p < 0.05).

Categorizing the HPV results according to cancer risk (Table **4**), 15.8% were positive for HPV16, 10.5% were positive for HPV18, and 26.3% were positive for HPV16 or HPV18; 11.1% tested positive for HPV genotypes not targeted by HC2. The percentage of HPV16 positives increased with increasing severity of disease (p_trend_ < 0.001), from negative histology (11.9%) to cancer (40.0%). A similar increase was not observed for HPV18, or for any other HPV genotype (data not shown). Concomitantly, the percentage of positives for carcinogenic HPV genotypes other than HPV16 and HPV18 decreased with increasing severity of disease (p_trend_ < 0.001). HPV16 and HPV18 accounted for up to 21.4% of CIN2, 46.2% of CIN3, and 80% of cancers. HPV16- and/or HPV18-positive CIN2/3 occurred at younger median age than CIN2/3 due to other HPV genotypes (37 years vs. 40.5 years, respectively, p = 0.06).

## Discussion

To our knowledge, this is the first report of carcinogenic HPV prevalence and HPV genotypes in cervical disease in women living in Haiti. This was a large community-based sample of nearly 10,000 women living in Port-au-Prince and Leogane, Haiti, resulting in precise estimates of carcinogenic HPV prevalence by age, which can be used to guide primary and secondary cervical cancer prevention strategies. 

**Figure 2:**
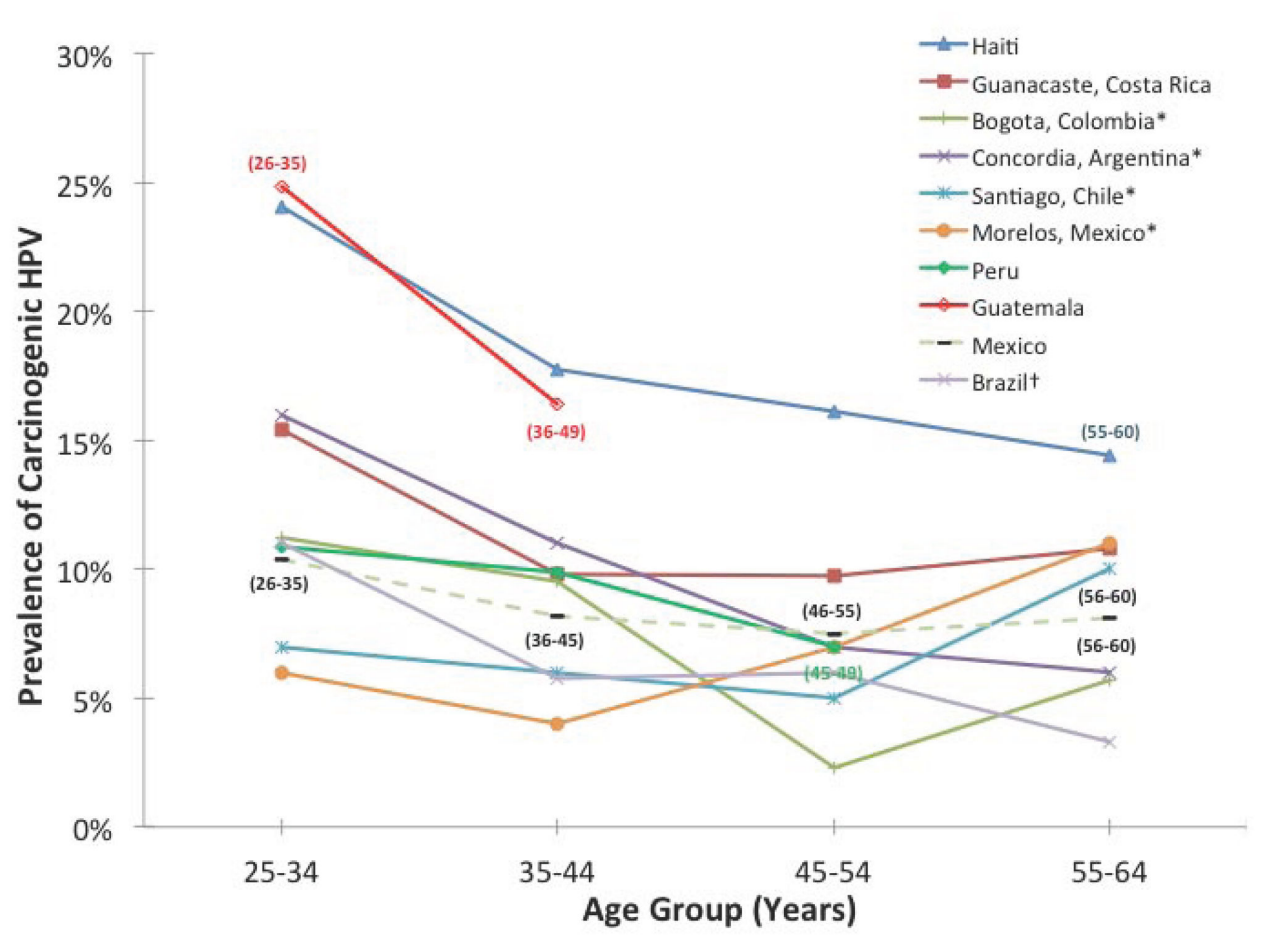
Age-range-specific carcinogenic HPV prevalence in Latin America compared to Haiti. A comparison of carcinogenic HPV prevalence for selected cross-sectional studies conducted in Latin America: Haiti (from this study); Guanacaste [16]; Colombia [17], Argentina [18], Chile [17], Mexico [17]; Peru [18]; Guatemala [20]; and a second study from Mexico [19]. *estimated from graphs; †Drs. Eduardo Franco and Luisa Villa, unpublished data, Ludwig-McGill Cohort, Brazil.

It is noteworthy that the prevalence of carcinogenic HPV was very high in this population compared to other populations in Latin America. As shown in Figure **2**, the age-specific carcinogenic HPV prevalence in this sample of Haitian women was significantly greater, in many cases two- or more fold greater, than the prevalence reported for populations from Costa Rica [16], Colombia [17], Argentina [18], Chile [17], Mexico [17], Peru [18], Brazil (Drs. Eduardo Franco and Luisa Villa, unpublished data, Ludwig-McGill Cohort, Brazil), and a second study in Mexico [19]. Only the carcinogenic HPV prevalence in Guatemala in a small sample of the general population [20] was comparable to the age-specific prevalence in Haiti. The age-specific prevalence of carcinogenic HPV in these Haitian women was more than twice that of immigrant Haitian women living in Little Haiti, Miami [21]. 

We observed that the age of diagnosis of HPV16/18-positive CIN2/3 was approximately three years younger than the diagnosis of CIN2/3 related to other carcinogenic HPV types. This is consistent with previous reports of HPV16- or HPV16/18-CIN2/3, CIN2, or CIN3 being diagnosed at an earlier age than CIN2/3 due to other HPV genotypes [22–26]. A similar phenomenon of type-dependent age of onset is seen in invasive cervical cancer [8,22,25].

One of the important limitations of this study was that only visible lesions were biopsied, undoubtedly leading to an under-ascertainment of disease; there were 42 CIN2+ (0.4%), 52 CIN3+ (0.5%), and 5 cancers (0.05%) (n.b., one symptomatic cancer tested HC2 negative; this was instantly diagnosed once the speculum was inserted, as the advanced lesion was large and easily visualized) diagnosed. It has been repeatedly shown that taking more biopsies, including the taking of biopsies of normal-appearing tissue, increases the diagnostic yield of colposcopy for CIN2+ and CIN3+ [27,28]. Of note, there was higher prevalence of HPV16 in women who did not have a biopsy taken compared to women who had a negative or CIN1 biopsy (16.3% vs. 11.3% or 11.5%, respectively). Speculatively, this would suggest that there was a significant number of undiagnosed CIN2+ in those who were not biopsied (reflected in the higher prevalence of HPV16), which was 70.5% of all HC2 positives.

We acknowledge several other limitations of this study. First, the prevalence of carcinogenic HPV genotypes was predicated on being HC2-positive and therefore any HPV genotype-specific biases of HC2 would influence these measurements. However, HC2 is clinically very sensitive for CIN2+ [29] so these biases are expected to be minimal. Second is that the age range that was used in screening this patient population was limited to women 25 and older, precluding an evidence-based description of genotypes in younger women and girls. However, we did not observe any age trends of HPV genotypes (among HC2 positives) and therefore would predict that the proportion of HPV16/18 to all HPV infections is similar at younger ages. We showed in Table **3** that women who started having sex at a younger age had a higher exposure to HPV, perhaps due to unmeasured risk behaviors linked to early initiation, emphasizing the importance of prophylactic HPV vaccination of the population before most have started having sex, i.e., ages 9-12 years. Finally, this was not a true population sample but an opportunistic sample of the population. Women were recruited to attend the free clinic and a percentage might have been motivated by pre-existing conditions; however, we note that HPV infection and precancerous disease are completely asymptomatic and therefore are unlikely to lead to a (biased) over-estimate of these conditions. Rather, we noted that the responses from participants suggested that the women were more interested in a free comprehensive health exam than in volunteering due to a pre-condition (unpublished observations).

In conclusion, we found a very high prevalence of carcinogenic HPV in this population. The high prevalence of carcinogenic HPV infection and the absence of cervical cancer screening may explain the high burden of cervical cancer in Haiti [2,3].
